# Anti-inflammatory protein TSG-6 secreted by bone marrow mesenchymal stem cells attenuates neuropathic pain by inhibiting the TLR2/MyD88/NF-κB signaling pathway in spinal microglia

**DOI:** 10.1186/s12974-020-1731-x

**Published:** 2020-05-11

**Authors:** Hao Yang, Lingmin Wu, Huimin Deng, Yuanli Chen, Huanping Zhou, Meiyun Liu, Shaochen Wang, Li Zheng, Lina Zhu, Xin Lv

**Affiliations:** 1 Department of Anesthesiology, Shanghai Pulmonary Hospital, Tongji University School of Medicine, 507 Zhengmin Rd, Shanghai, 200433 China; 2 Department of Anesthesiology, The first Hospital of Anhui Medical University, 218 Jixi Rd, Hefei, 230022 China; 3 Department of Anesthesiology, Fuyang Hospital of Anhui Medical University, 99 Huangshan Rd, Fuyang, 236000 China

**Keywords:** Neuropathic pain, BMSCs, TSG-6, Neuroinflammation, TLR2, Microglia

## Abstract

**Background:**

Neuroinflammation plays a vital role in the development and maintenance of neuropathic pain. Recent evidence has proved that bone marrow mesenchymal stem cells (BMSCs) can inhibit neuropathic pain and possess potent immunomodulatory and immunosuppressive properties via secreting a variety of bioactive molecules, such as TNF-α-stimulated gene 6 protein (TSG-6). However, it is unknown whether BMSCs exert their analgesic effect against neuropathic pain by secreting TSG-6. Therefore, the present study aimed to evaluate the analgesic effects of TSG-6 released from BMSCs on neuropathic pain induced by chronic constriction injury (CCI) in rats and explored the possible underlying mechanisms in vitro and in vivo.

**Methods:**

BMSCs were isolated from rat bone marrow and characterized by flow cytometry and functional differentiation. One day after CCI surgery, about 5 × 10^6^ BMSCs were intrathecally injected into spinal cerebrospinal fluid. Behavioral tests, including mechanical allodynia, thermal hyperalgesia, and motor function, were carried out at 1, 3, 5, 7, 14 days after CCI surgery. Spinal cords were processed for immunohistochemical analysis of the microglial marker Iba-1. The mRNA and protein levels of pro-inflammatory cytokines (IL-1β, TNFα, IL-6) were detected by real-time RT-PCR and ELISA. The activation of the TLR2/MyD88/NF-κB signaling pathway was evaluated by Western blot and immunofluorescence staining. The analgesic effect of exogenous recombinant TSG-6 on CCI-induced mechanical allodynia and heat hyperalgesia was observed by behavioral tests. In the in vitro experiments, primary cultured microglia were stimulated with the TLR2 agonist Pam3CSK4, and then co-cultured with BMSCs or recombinant TSG-6. The protein expression of TLR2, MyD88, p-p65 was evaluated by Western blot. The mRNA and protein levels of IL-1β, TNFα, IL-6 were detected by real-time RT-PCR and ELISA. BMSCs were transfected with the TSG-6-specific shRNA and then intrathecally injected into spinal cerebrospinal fluid in vivo or co-cultured with Pam3CSK4-treated primary microglia in vitro to investigate whether TSG-6 participated in the therapeutic effect of BMSCs on CCI-induced neuropathic pain and neuroinflammation.

**Results:**

We found that CCI-induced mechanical allodynia and heat hyperalgesia were ameliorated by intrathecal injection of BMSCs. Moreover, intrathecal administration of BMSCs inhibited CCI-induced neuroinflammation in spinal cord tissues. The analgesic effect and anti-inflammatory property of BMSCs were attenuated when TSG-6 expression was silenced. We also found that BMSCs inhibited the activation of the TLR2/MyD88/NF-κB pathway in the ipsilateral spinal cord dorsal horn by secreting TSG-6. Meanwhile, we proved that intrathecal injection of exogenous recombinant TSG-6 effectively attenuated CCI-induced neuropathic pain. Furthermore, in vitro experiments showed that BMSCs and TSG-6 downregulated the TLR2/MyD88/NF-κB signaling and reduced the production of pro-inflammatory cytokines, such as IL-1β, IL-6, and TNF-α, in primary microglia treated with the specific TLR2 agonist Pam3CSK4.

**Conclusions:**

The present study demonstrated a paracrine mechanism by which intrathecal injection of BMSCs targets the TLR2/MyD88/NF-κB pathway in spinal cord dorsal horn microglia to elicit neuroprotection and sustained neuropathic pain relief via TSG-6 secretion.

## Background

Neuropathic pain is characterized as a hypersensitive response to noxious and innocuous stimuli that results from primary injury or dysfunction of the somatosensory nervous system; it can persist for months to years, even after the primary tissue damage has healed [1]. Neuropathic pain remains as a chronic debilitating condition that affects the quality of life and reduces individual productivity; it is estimated to affect up to 10% of the population [2, 3]. The etiology of neuropathic pain is complex and includes diabetic neuropathy, spinal cord injury, post-herpetic neuralgia, demyelinating disease, and cancer. In the past, various therapies for neuropathic pain, including pharmacotherapy [4], nerve block [5], spinal cord simulation [6], neurosurgical lesioning, and surgery [7], have been used. However, these treatment methods are not fully effective in relieving neuropathic pain.

Growing evidence suggests that neuroinflammation and the immune system contribute importantly to the development and maintenance of neuropathic pain [8, 9]. Neuroinflammation results from the activation of glial cells, including satellite glial cells, microglia, and astrocytes, in the peripheral and central nervous system as well as the activation of immune cells including resident mast cells, infiltrating macrophages, and neutrophils [10–13]. Microglia, the main immunocompetent cells in the central nervous system (CNS) that regulate homeostasis in the brain and spinal cord, represent 5–10% of the glia in the CNS. Numerous studies have shown that activated microglia in the spinal dorsal horn play a key role in nerve injury-induced or cancer-related neuropathic pain [14–18]. Activated microglia change their morphology from a resting, ramified shape into an active, amoeboid shape and exacerbate neuropathic pain by releasing pro-inflammatory cytokines, such as tumor necrosis factor (TNF)-α, interleukin (IL)-1β, IL-6, and chemokines to activate and sensitize spinal cord nociceptive neurons [19–21].

Toll-like receptors (TLRs) are evolutionarily conserved pattern recognition receptors that can mediate innate and adaptive immunity against exogenous or endogenous dangerous ligands and danger-associated molecular patterns produced after tissue injury [22]. In glial cells, activation of TLRs produces a wide range of pro-inflammatory cytokines, including interleukin-1β (IL-1β), tumor necrosis factor-α (TNF-α), and IL-6. Previous studies have found that TLR2 is widely expressed in the CNS and contributes to the nerve injury-induced spinal cord glial cell activation and is necessary for the development of neuropathic pain [23–26]. The myeloid differentiation factor-88 adaptor protein (MyD88) is involved in TLR2 signaling and leads to NF-κB activation, which results in the expression of NF-κB-targeted genes, such as TNF-α and IL-1β [27].

Recently, cell-based therapy has been developed as a potential strategy for regulating inflammation and repairing tissue injuries. Bone marrow mesenchymal stem cells (BMSCs) are multi-potent stem cells with relatively low immunogenicity and have emerged as a novel and promising candidate for therapeutic intervention for a variety of inflammatory diseases, such as sepsis, lung injury, and acute pancreatitis [28–30]. BMSCs are easy to isolate from healthy donors or patients, expand rapidly in culture, and differentiate into several cellular phenotypes in vitro and in vivo. The original assumption in the exploration of the mechanisms by which BMSCs repair damaged tissues was that they migrate to injured tissues and differentiate to replace injured cells. However, recent studies have revealed that the protective effects of mesenchymal stem cells might be due to potent immunomodulatory and immunosuppressive properties via the secretion of a variety of bioactive molecules, such as growth factors and anti-inflammatory molecules [31–33]. Studies have proven that BMSCs can alleviate neuropathic pain by paracrine secretion [34–37]; however, the precise mechanisms of this action remain ambiguous. The anti-inflammatory protein TNF-α-stimulated gene 6 protein (TSG-6) is a 35–38 kD glycoprotein expressed in a variety of cell types in response to pro-inflammatory cytokines [38, 39]. Previous studies have shown that BMSCs can secrete TSG-6 to modulate the inflammatory microenvironment partly by inhibiting the activation of the TLR2/NF-κB signaling pathway [40, 41]. Recently, we also proved that TSG-6 secreted by BMSCs can delay or even prevent intervertebral disc degeneration partly by suppressing the activation of the TLR2/MyD88/NF-κB signaling pathway in nucleus pulposus cells [27]. However, whether TSG-6 secreted by BMSCs exerts an anti-nociceptive effect in neuropathic pain by directly inhibiting the activation of the TLR2/MyD88/NF-κB signaling pathway in spinal dorsal horn microglia remains unclear. Therefore, in the present study, we examined the anti-nociceptive effects of BMSCs on CCI-induced neuropathic pain and investigated whether TSG-6 secreted from BMSCs exerts its protective effect by suppressing the activation of the TLR2/MyD88/NF-κB signaling pathway and attenuating neuroinflammation in spinal dorsal horn microglia.

## Methods

### Ethics statement

All experiments and surgical procedures were approved by the Animal Care and Use Committee of the Tongji University School of Medicine, adhered to the recommendations in the Guide for the Care and Use of Laboratory Animals published by the National Institutes of Health, and complied with the relevant sections of the ARRIVE guidelines.

### Reagents

Dulbecco’s modified Eagle’s medium (DMEM), fetal bovine serum (FBS), streptomycin, phosphate-buffered saline (PBS), ethylenediaminetetraacetic acid (EDTA), α-MEM, and DMEM/F12 were purchased from Gibco (Grand Island, NY, USA). Anti-CD11b-PE, anti-CD29-PE, anti-CD-34-FITC, anti-CD45-PE, anti-CD90-PE, anti-CD105 PE, and isotype control antibody were purchased from eBioscience (San Diego, CA, USA). Dexamethasone, indomethacin, insulin, 3-isobutyl-1-methyl-xanthine, transferrin, sodium pyruvate, β-glycerophosphate, l-ascorbic acid 2-phosphate, and DAPI were purchased from Sigma-Aldrich (St. Louis, MO, USA). PrimeScript RT Master Mix was purchased from Takara (DaLian, China). iTaq universal SYBR Green Supermix was purchased from Bio-Rad (Hercules, CA, USA). Bicinchoninic acid (BCA) protein assay kit was purchased from Thermo Scientific (Rockford, IL, USA). Anti-IL-1β, anti-IL-6, anti-TNF-α, anti-Iba-1, USA), anti-TLR2, anti-MyD88, anti-GFAP, anti- IBA-1, anti-NeuN were purchased from Abcam (Cambridge, UK). Anti-p65, anti-phospho-p65, and anti-β-actin were purchased from Cell Signaling Technology (Beverly, MA, USA). Anti-TLR2 was purchased from Santa Cruz Biotechnology (Santa Cruz, CA, USA). Alexa Fluor 488-conjugated donkey anti-mouse IgG and Alexa Fluor 594-conjugated donkey anti-rabbit IgG were purchased from Jackson Immunoresearch (West Grove, PA, USA). Recombinant Human TSG-6 Protein was purchased from R&D systems (Minneapolis, MN, USA). Lentivirus-TSG-6-shRNA or NC-shRNA were purchased from Huzbio biotechnology (Shanghai, China).

### Animals

Surgical procedures were performed on adult male Sprague-Dawley (SD) rats weighing 200–250 g. All rats were housed in a standard animal care room with a 12-h/12-h light/dark cycle and had free access to food and water.

### Isolation, culture, and identification of BMSCs

SD rat primary BMSCs were prepared as previously described [27]. Briefly, primary BMSCs were obtained from the bone marrow of the tibias and femurs of 4-week-old male SD rats under aseptic conditions. A syringe fitted with an 18-gauge needle was inserted into the bone marrow cavity, and then the bone marrow was flushed out with 5 ml of DMEM/F12 (Gibco, Grand Island, NY, USA) supplemented with 10% fetal bovine serum (FBS; Gibco), 100 U/ml penicillin, and 100 μg/ml streptomycin (Gibco). The cells were placed in 25 cm^2^ culture flasks (Corning, NY, USA) and cultured at 37 °C under 5% CO_2_ and 90% humidity. After 48-h of incubation, the non-adherent cells were removed and fresh culture medium was added to the flasks. The medium was changed every 3 days. When the cells reached 90% confluence, the adherent cells were washed with phosphate-buffered saline (PBS) and harvested by incubation with 1 ml of 0.25% trypsin and 1 mM ethylenediaminetetraacetic acid (EDTA) for 1 min at 37 °C. The trypsin was neutralized with 5 ml of complete medium and the cells were passaged at a dilution of 1:3. BMSCs at passage 3 were used for all experiments. BMSCs were assessed using flow cytometry to detect cells that expressed typical markers; the antibodies were as follows: CD11b-PE, CD29-PE, CD-34-FITC, CD45-PE, CD90-PE, CD105 PE, and isotype control (eBioscience, San Diego, CA, USA).

### BMSCs differentiation assays

Prior to using BMSCs for in vitro and in vivo experiments, we examined their multilineage differentiation under adipogenic, chondrogenic, and osteogenic differentiation conditions using oil red O staining (Sigma-Aldrich, St. Louis, MO, USA), alcian blue staining (Sigma-Aldrich), and alizarin red staining (Sigma-Aldrich), respectively.

BMSCs were seeded into 6-well plates (Corning) at 2 × 10^5^ cells/well and cultured until they reached confluence. For adipogenic differentiation analysis, cells were incubated in adipogenic induction medium α-MEM (Gibco) containing 10% FBS (Gibco), 1 mmol/L dexamethasone (Sigma-Aldrich), 0.2 mmol/L indomethacin(Sigma-Aldrich), 10 mg/ml insulin(Sigma-Aldrich), and 0.5 mmol/L 3-isobutyl-1-methyl-xanthine (Sigma-Aldrich) for 21 days. The adipogenic induction medium was changed every 3 days. After 21 days of induction, the cells were fixed in PBS containing 10% formaldehyde solution and stained with oil red O. For chondrogenic differentiation analysis, cells were treated with chondrogenic induction medium low-glucose DMEM (Gibco) supplemented with 2 mg/L insulin (Sigma-Aldrich), 3 mg/L transferrin (Sigma-Aldrich), 1 mmol/L sodium pyruvate (Sigma-Aldrich), 100 nmol/L dexamethasone (Sigma-Aldrich), and 10 μg/L transforming growth factor β1 (TGF-β1; PeproTech, USA). The chondrogenic induction medium was changed every 3 days. After 28 days of induction, the cells were then stained with alcian blue. For osteogenic differentiation analysis, cells were incubated in α-MEM (Gibco) supplemented with 10% FBS (Gibco), 100 nmol/L dexamethasone (Sigma-Aldrich), 10 mmol/L β-glycerophosphate (Sigma-Aldrich), and 50 μmol/L L-ascorbic acid 2-phosphate (Sigma-Aldrich). The medium was replaced every 3 days. After 28 days of induction, the mineralized osteocytes were visualized with alizarin red staining.

### Surgical protocol

The chronic constriction injury (CCI) model was induced in rats as previously described by Chen et al. [42]. Briefly, male rats were anesthetized with i.p. sodium pentobarbital (40–60 mg/kg) and adequate anesthesia was ascertained by a lack of response to a nociceptive stimulus. The left common sciatic nerve was exposed at the mid-thigh level, and four 4–0 chromic gut sutures were tied loosely around the nerve at approximately 1-mm intervals. This approach ensured that circulation through the epineural vasculature was preserved. The sham group underwent surgery without making ligation after sciatic nerve exposure.

### Intrathecal catheter implantation

For intrathecal injection in rats, under sodium pentobarbital anesthesia, a 32-gauge intrathecal catheter (ReCathCo, Allison Park, PA, USA) was inserted through the atlanto-occipital membrane into the lumbar enlargement and externalized through the skin. Subsequently, the catheter was placed under the skin from the lower back to the head. To verify whether the catheter was inserted successfully, 2% lidocaine (20 μl) was given intrathecally.

### BMSCs or TSG-6 treatment

The rats were randomly divided into the following groups: the sham group; the CCI group; the CCI + PBS group; the CCI + BMSCs (5 × 10^5^) group; the CCI + BMSCs (1 × 10^6^) group; the CCI + BMSCs (5 × 10^6^) group; the CCI + TSG-6-shRNA-BMSCs group; the CCI + NC-shRNA-BMSCs group; the CCI + 1 μg TSG-6 group; the CCI + 5 μg TSG-6 group. Sham group: rats exposed left common sciatic nerve but not ligated. CCI group: rats underwent CCI surgery. CCI + PBS group: rats underwent CCI surgery and were then intrathecally injected with 10 μl of PBS on day 1 after CCI surgery. CCI + BMSCs (5 × 10^5^) group: rats underwent CCI surgery and were then intrathecally injected with 5 × 10^5^ BMSCs in 10 μl of PBS on day 1 after CCI surgery. CCI + BMSCs (1 × 10^6^) group: rats underwent CCI surgery and were then intrathecally injected with 1 × 10^6^ BMSCs in 10 μl of PBS on day 1 after CCI surgery. CCI + BMSCs (5 × 10^6^) group: rats underwent CCI surgery and were then intrathecally injected with 5 × 10^6^ BMSCs in 10 μl of PBS on day 1 after CCI surgery. CCI + TSG-6-shRNA-BMSCs group: rats underwent CCI surgery and were then intrathecally injected with 5 × 10^6^ TSG-6-shRNA-BMSCs in 10 μl of PBS on day 1 after CCI surgery. CCI + NC-shRNA-BMSCs group: rats underwent CCI surgery and were then intrathecally injected with 5 × 10^6^ NC-shRNA-BMSCs in 10 μl of PBS on day 1 after CCI surgery. CCI + 1 μg TSG-6 group: rats underwent CCI surgery and were then intrathecally injected with 10 μl of recombination TSG-6 (1  μg) on day 7 after CCI surgery. CCI + 5 μg TSG-6 group: rats underwent CCI surgery and then intrathecally injected with 10 μl of recombination TSG-6 (5 μg) on day 7 after CCI surgery.

### Behavioral tests

Rats were habituated to the testing environment for 3 days before baseline testing. The behaviors of all rats were tested blindly. Paw withdrawal thresholds (PWT) was considered as mechanical allodynia. PWT was measured by stimulating the mid-plantar surface of the hind paw with a Dynamic Plantar Aesthesiometer (Ugo Basile, Comerio, Italy). The rats were placed in a test cage with a wire mesh floor, and a thin rod with a diameter of 0.5 mm was applied to the skin of the midplantar area of the hind paw. The filament exerted an increasing force ranging from 0 to 50 g over a period of 20 s at a rate of 2.5 g/s. When the animal withdrew its hind paw, the steel rod stimulus was halted, and the force at which the animal withdrew its hind paw was recorded. The mean of five consecutive trials with an interval of at least 5 min was used for analysis.

Paw withdrawal latency (PWL) was considered as thermal hyperalgesia. PWL was tested using a Hargreaves radiant heat apparatus (IITC Life Science, Woodland Hills, CA, USA) with the basal paw withdrawal latency adjusted to 10 to 14 s and a cutoff of 20 s to prevent tissue damage. The stimulation was applied five times with an interval of at least 10 min.

A rotarod system (IITC Life Science) was used to assess motor function. Rats were tested in three trials separated by 15-min intervals. The rotation started at a speed of 5 rpm and accelerated constantly up to a maximum speed of 20  rpm within 300 s. The fall latency was recorded and averaged.

### Microglial culture

Primary cultured microglial cells were prepared as described previously [43]. In brief, a mixed glial culture was prepared from the cerebral cortex of neonatal SD rats and maintained in Dulbecco’s modified Eagle’s medium (DMEM) with 10% fetal bovine serum (FBS). The medium was changed every 3 days. After 12 to 14 days, microglial cells were isolated from the mixed glial culture as floating cells by a gently shaking the culture flasks and the resulting cell suspension was transferred to plastic dishes for subsequent experiments. The purity of microglia reached approximately 97%, as determined by immunostaining for Iba-1.

### Transwell co-culture of microglia with BMSCs and TSG-6

A 6-well transwell system (0.4 μm pore size membrane; Corning) was used to assess the effects of BMSCs and TSG-6 on primary microglia that had been stimulated using the TLR2 specific agonist Pam3CSK4 (InvivoGen, San Diego, CA, USA). A total of 2 × 10^5^ microglia were placed in the lower chamber, and 1 day later the microglia were either treated with 400 ng/ml of Pam3CSK4 or left untreated for 1 h. Subsequently, these microglia were co-cultured with one of the following treatments in the upper chamber for 24 h: (1) control medium, (2) 2.0 × 10^5^ BMSCs, (3) 2.0 × 10^5^ BMSCs transfected with TSG-6-shRNA, (4) 2.0 × 10^5^ BMSCs transfected with negative control (NC)-shRNA, (5) recombinant TSG-6 at 200 ng/ml, (6) recombinant TSG-6 at 400 ng/ml, or (7) recombinant TSG-6 at 600 ng/ml.

### Real-time RT-PCR

Total RNA was isolated from the lumbar segment of the ipsilateral spinal dorsal horn or primary microglia using a RNeasy Mini Kit (QIAGEN) according to the manufacturer’s instructions. cDNA synthesis was performed using PrimeScript RT Master Mix (Takara, China), and real-time RT-PCR was performed on a LightCycler 480 real-time PCR system (Roche, USA) using iTaq universal SYBR Green Supermix (Bio-Rad, Hercules, CA, USA). β-Actin was employed as the endogenous control. The primer sequences were designed using Primer 5.0 software and are listed in Table 1. The data are presented as relative Ct values. The 2^-ΔΔCt^ method was employed to calculate relative expression levels.

**Table 1:** Sequences of primers used in real-time RT-PCR

mRNA	Primers	Sequences (5′-3′)
TSG-6	Upstream	AGGCTGTTTGGCTGACTATGT
	Downstream	TTTCCTGTGCTGATGATGTCTT
IL-1β	Upstream	TGCTGATGTACCAGTTGGGG
	Downstream	CTCCATGAGCTTTGTACAAG
IL-6	Upstream	GCCCTTCAGGAACAGCTATG
	Downstream	CAGAATTGCCATTGCACAAC
TNF-α	Upstream	TGATCGGTCCCAACAAGGA
	Downstream	TGCTTGGTGGTTTGCTACGA
β-actin	Upstream	TCAGGTCATCACTATCGGCAAT
	Downstream	AAAGAAAGGGTGTAAAACGCA

### Western blot analysis

Total protein from the lumbar segment of the ipsilateral spinal dorsal horn or primary microglia was extracted using RIPA lysis buffer containing a protease inhibitor, a phosphatase inhibitor, and PMSF (Beyotime, Shanghai, China). Protein concentrations were estimated using a bicinchoninic acid (BCA) protein assay kit (Thermo Scientific, Rockford, IL, USA). The proteins were separated by SDS-PAGE and transferred to a PVDF membrane (Millipore, Billerica, MA, USA). The membranes were blocked with Tris-buffered saline and Tween 20 (TBST) containing 5% non-fat dry milk and then incubated overnight at 4 °C in the presence of primary antibodies against IL-1β (1:2000 dilution; Abcam, USA), IL-6 (1:4000 dilution; Abcam, USA), TNF-α (1:2000 dilution; Abcam, USA), Iba-1 (1:2000 dilution; Abcam, USA), TLR2, MyD88 (1:1000 dilution; Abcam, USA), p65(1:2000 dilution; Cell Signaling Technology, USA), phospho-p65 (1:1000 dilution; Cell Signaling Technology, USA), TSG-6 (1:500 dilution; Santa Cruz, USA), and β-actin (1:8000 dilution; Cell Signaling Technology, USA). The secondary antibodies were applied for 1 h at room temperature. The immunoblots were visualized using enhanced chemiluminescence (ECL; Thermo Scientific), and relative protein concentrations were measured using Quantity One software (Bio-Rad). The expression level of β-actin was used as an internal control.

### Immunofluorescence

For the in vivo study, rats were deeply anesthetized with isoflurane and perfused through the ascending aorta with 0.9% saline solution, followed by 4% paraformaldehyde. Immediately after perfusion, the spinal cord was isolated via hydro-extrusion and post-fixed overnight in 4% paraformaldehyde at 4 °C. The tissues were cryoprotected by incubation in 20% sucrose solution for 24 h at 4 °C, followed by incubation in 30% sucrose solution for 48 h at 4 °C. The L4-L5 segments of the spinal cord were then immediately removed and cut with a cryostat into 20-μm-thick sections. The sections were incubated overnight at 4 °C with the following primary antibodies: mouse GFAP (1:300 dilution; Abcam, USA); goat IBA-1(1:200 dilution; Abcam, USA); mouse NeuN (1:100 dilution; Abcam, USA); and rabbit TLR2 (1:100; Santa Cruz, USA). The sections were subsequently incubated for 1 h at room temperature with Alexa Fluor 488-conjugated donkey anti-goat IgG (1:500; Jackson Immunoresearch, USA), Alexa Fluor 488-conjugated donkey anti-mouse IgG (1:500; Jackson Immunoresearch, USA), and Alexa Fluor 594-conjugated donkey anti-rabbit IgG (1:500; Jackson Immunoresearch, USA). DAPI (Sigma-Aldrich, USA) was used to stain the cell nuclei. Stained sections were observed by confocal microscopy (Olympus, Tokyo, Japan).

For the in vitro study, a total of 2 × 10^5^ microglia were grown on glass slides placed in the lower chamber, and the microglia cells were treated with 400 ng/ml Pam3CSK4 or left untreated for 1 h. Subsequently, these microglia cells were co-cultured with one of the following treatments in the upper chamber: (1) control medium, (2) 2.0 × 10^5^ BMSCs, (3) 2.0 × 10^5^ BMSCs transfected with TSG-6-shRNA, (4) 2.0 × 10^5^ BMSCs transfected with NC-shRNA, or (5) these microglia cells were treated with recombinant TSG-6 protein (200 ng/ml, 400 ng/ml, 600 ng/ml, respectively). After 24 h of transwell co-culture, the cells were washed with PBS and fixed with freshly prepared 4% paraformaldehyde for 15 min at room temperature and then washed three times with PBS. Each cover slip was then blocked in 5% BSA and subsequently incubated with a rabbit NF-κB p65 antibody (1:200 dilution; Cell Signaling Technology, USA) overnight at 4 °C. The next day, all samples were washed with PBS and incubated with Alexa Fluor 488-conjugated donkey anti-rabbit IgG (1:500; Jackson Immunoresearch, USA) for 1 h at room temperature. After the samples were washed with PBS, DAPI (Sigma-Aldrich) was used to stain the cell nuclei. The slides were visualized by confocal microscopy (Olympus, Tokyo, Japan) and analyzed using ImageJ software.

### Transfection of BMSCs with shRNA

BMSCs at passage 3 were used for transfection. Briefly, BMSCs were plated at 2 × 10^5^ cells per well in 6-well dishes and cultured for 24 h. The cells were then transfected with lentivirus-TSG-6-shRNA or NC-shRNA (Huzbio, Shanghai, China) according to the manufacturer’s instructions. To confirm the knockdown of TSG-6, we extracted RNA and protein from aliquots of the cells and analyzed TSG-6 expression using real-time RT-PCR and Western blot (Table 2).

**Table 2:** Sequences of shRNA used in BMSCs transfection

shRNA	Sequences (5′-3′)
shRNA-1	GCAGCAGGCGTATACCATAGA
shRNA-2	GCTGGCAGATACAAGCTAACC
shRNA-3	GCATCATTGATTATGGAATCC

### Enzyme-linked immunosorbent assay

Enzyme-linked immunosorbent assay (ELISA) was performed using microglial culture medium and L4–L5 spinal cords ipsilateral to the nerve injury. Spinal cord tissues were homogenized in lysis buffer containing protease and phosphatase inhibitors, incubated on ice for 5  min, sonicated, and cleared by centrifugation (15,000 g for 15 min at 4 °C). The supernatants were collected and protein content was determined using a MicroBCA assay (Thermo Scientific, Germany). IL-1β, IL-6, and TNF-α were measured using the respective ELISA kit (R&D Systems, Wiesbaden, Germany) according to the manufacturer’s instructions. TSG-6 was measured using the ELISA kit (Cusabio, Wuhan, China) according to the manufacturer’s instructions.

### Trafficking of BMSCs to the spinal dorsal horn

To examine the distribution of transplanted CM-Dil–labeled BMSCs following intrathecal injection, lumbar spinal cord segments were collected. In brief, rats were deeply anesthetized with isoflurane and perfused through the ascending aorta with 0.9% saline solution, followed by 4% paraformaldehyde. Immediately after perfusion, the spinal cord was isolated via hydro-extrusion and post-fixed overnight in 4% paraformaldehyde at 4 °C. The tissues were cryoprotected by incubation in 20% sucrose solution for 24 h at 4 °C followed by incubation in 30% sucrose solution for 48 h at 4 °C. The L4-L5 segments of the spinal cord were then immediately removed and cut with a cryostat into 20-μm-thick sections. After the samples were washed with PBS, DAPI (Sigma-Aldrich) was used to stain the cell nuclei. All sections were observed by confocal microscopy (Olympus, Tokyo, Japan).

### Statistical analyses

All experiments were performed at least three times, and the data are expressed as the mean value ± standard deviation (SD). Statistical analyses were performed using SPSS 17.0 software (SPSS, Chicago, Illinois, USA). Two-way analysis of variance (ANOVA) with post hoc Tukey test was used to compare the behavioral data of the different groups. Student’s *t* test (two-tailed) was used for comparisons between two groups. One-way analysis of variance (ANOVA) with post hoc Tukey test was used for the statistical analyses in other tests. Significance was set at a level of *P <* 0.05.

## Results

### Characterization of BMSCs

**Fig. 1:**
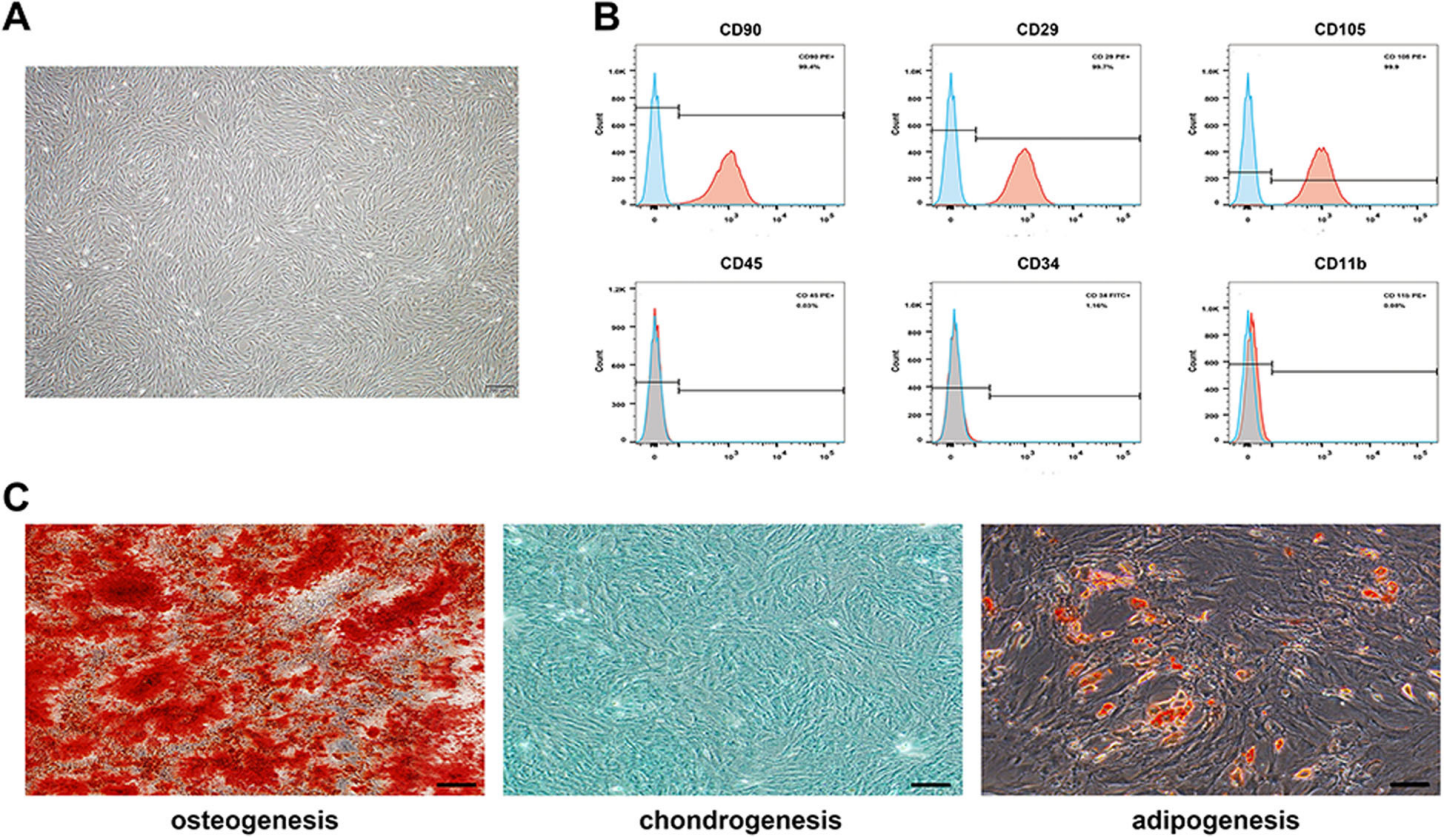
Characterization of BMSCs. Morphological observation of rat BMSCs (**a**) used in this study. Scale bar: 200 μm. **b** Flow cytometric analysis of BMSCs surface markers. The cells were positive for CD90, CD29, and CD105, and negative for CD45, CD34, and CD11b. **c** The multidifferentiation potential of BMSCs in vitro. Alizarin red S staining was used to evaluate osteogenic differentiation, Alcian blue staining was used to evaluate chondrogenic differentiation, and oil Red O staining was used to evaluate adipogenic differentiation. Scale bar: 30 μm

Primary cultured BMSCs adhered to the culture dish after approximately 24 h and reached 90% confluence within 7 days. BMSCs exhibited a flattened and spindle shape, which was similar to fibroblastic morphology (Fig. 1a). Flow cytometry analysis was used to identify a series of cell surface antigens on BMSCs at passage 3. Flow cytometry analysis showed that the cells were positive for the mesenchymal stem cell markers CD29, CD90, and CD105 and negative for the hematopoietic markers CD45, CD34, and CD11b (Fig. 1b). The in vitro differentiation model revealed that the BMSCs successfully differentiated into osteogenic, chondrogenic, or adipogenic lineages under specific differentiation induction (Fig. 1c).

### Intrathecal treatment with BMSCs ameliorated mechanical allodynia and thermal hyperalgesia induced by CCI

**Fig. 2:**
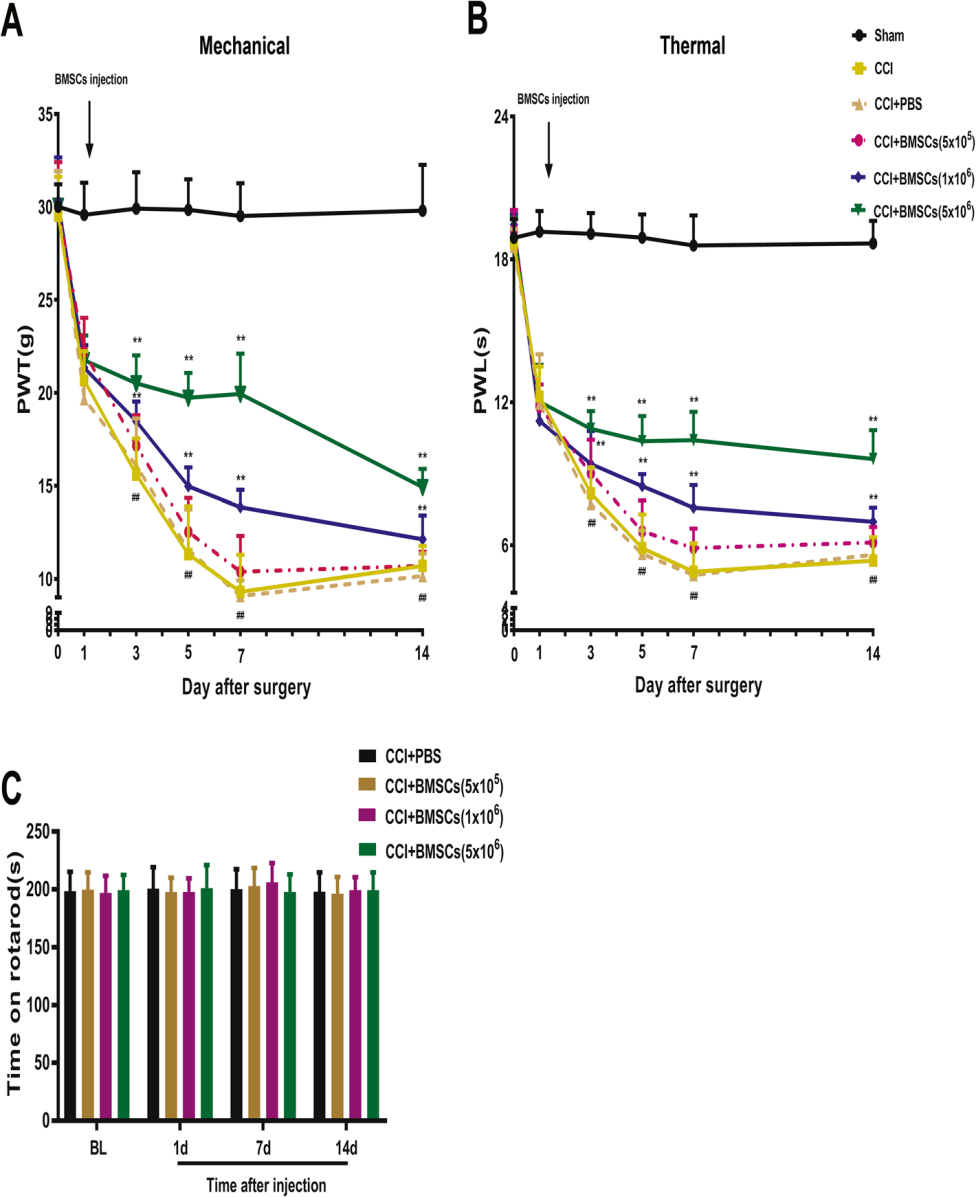
Inhibition of CCI-induced neuropathic pain in rats by a single intrathecal injection of BMSCs. Paw withdrawal mechanical threshold (**a**) and paw withdrawal thermal latency (**b**) were reduced after CCI in rats. In contrast, early intrathecal treatment (1 day after sham or CCI surgery) with BMSCs recovered mechanical allodynia and thermal hyperalgesia in a dose-dependent manner in CCI rats. **c** Rotarod test for the evaluation of motor function. Arrows in (**a**, **b**) indicated the time of BMSCs injection. The data are expressed as the means ± SD (*n* = 8 in each group). **P < 0.01 versus the CCI group; ^##^*P* < 0.01 versus the Sham group. Statistical significance was determined by two-way analysis of variance (ANOVA) with post hoc Tukey test

We induced neuropathic pain in rats via sciatic nerve chronic constriction injury (CCI) model. The results revealed that evident mechanical allodynia and thermal hyperalgesia developed within 1 day and was persistently maintained until day 14 after CCI compared with the sham group (Fig. 2a, b). To test the hypothesis that BMSCs alleviate neuropathic pain, we intrathecally injected BMSCs into the spinal cerebrospinal fluid (CSF) 1 day after CCI surgery, when neuropathic pain had developed but had not reached its peak. As shown in Fig. 2a, b, a single intrathecal injection of BMSCs significantly inhibited CCI-induced mechanical allodynia and thermal hyperalgesia in a dose-dependent manner, and this inhibited lasted for at least 14 days. However, intrathecal administration of PBS had no significant antinociceptive effects on CCI-induced mechanical allodynia or thermal hyperalgesia (Fig. 2a, b). Furthermore, BMSCs treatment did not affect motor function in any of the test groups, as evaluated by the rotarod test (Fig. 2c).

### Intrathecal administration of BMSCs inhibited neuroinflammation and suppressed microglial activation in the spinal cord after CCI

**Fig. 3:**
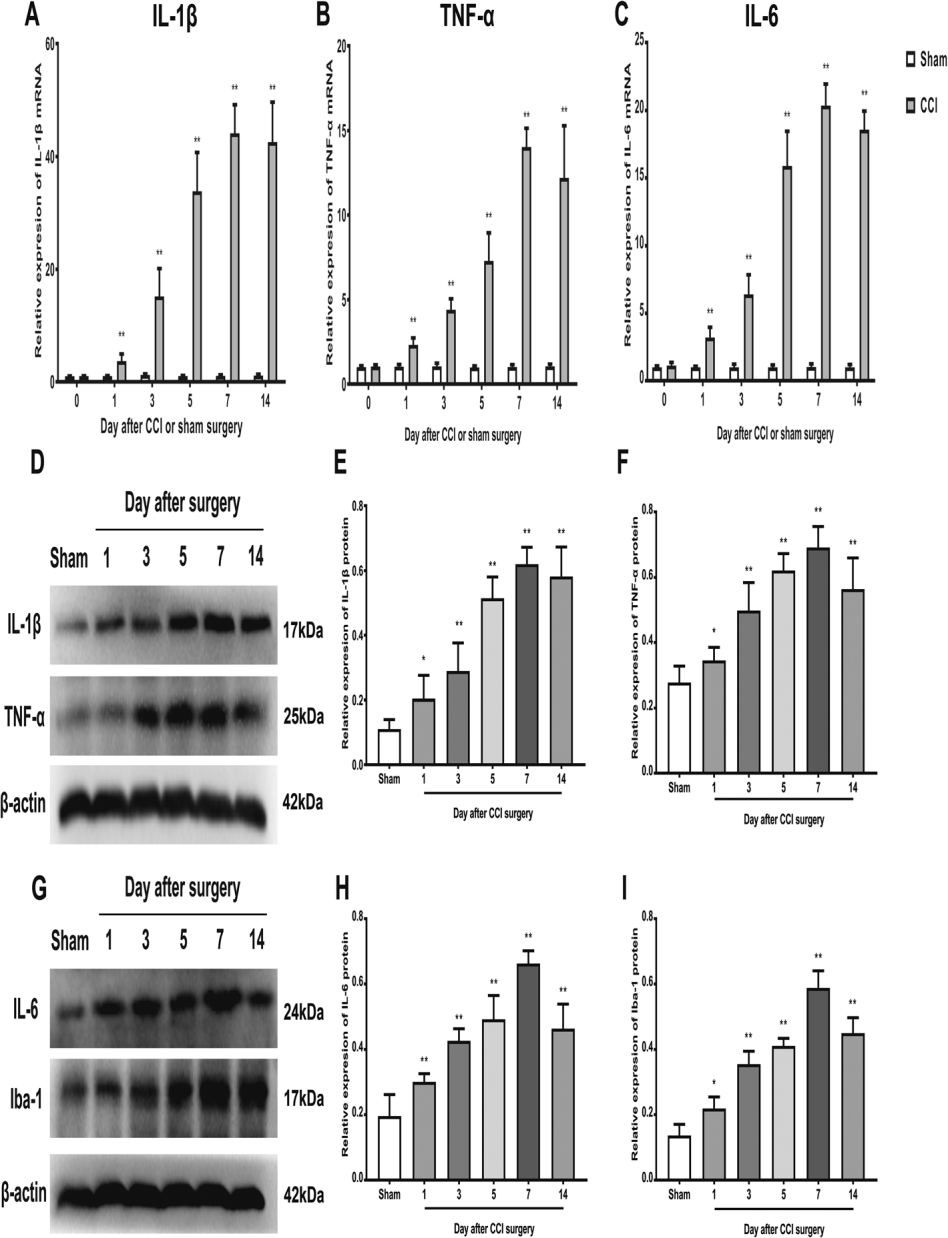
Neuropathic pain up-regulated the expression of pro-inflammatory cytokines and induced microglia activation in rat spinal cord dorsal horn. Real-time RT-PCR showed that CCI surgery significantly increased the expression of IL-1β (**a**), TNF-α (**b**), and IL-6 (**c**) in the ipsilateral spinal cord dorsal horn at each time point. All mRNA levels were normalized to the level of β-actin mRNA. **d**, **g** Representative image of protein levels of IL-1β, TNF-α, IL-6, and Iba-1. Quantitative analysis of Western blotting results showed that CCI surgery significantly increased the expression of IL-1β (**e**), TNF-α (**f**), IL-6 (**h**), and Iba-1 (**i**) in the ipsilateral spinal cord dorsal horn at each time point. The data are expressed as the means ± SD (*n* = 8 in each group). *P < 0.05; **P < 0.01 versus the sham group. Statistical significance was determined by one-way analysis of variance (ANOVA) with post hoc Tukey test

As neuroinflammation plays a key role in the progression of neuropathic pain, we examined the mRNA and protein expression of IL-1β, IL-6, and TNF-α in the ipsilateral spinal cord dorsal horn. As shown in Fig. 3a–h, the mRNA and protein expression of IL-1β, IL-6, and TNF-α in the ipsilateral spinal cord dorsal horn was significantly upregulated in a time-dependent manner in the CCI group compared with the sham group. Microglia, the resident immune cells of the CNS, are the predominant cells used to assess neuroinflammation. We next investigated the activation of microglia in the ipsilateral spinal cord dorsal horn. Our data showed that the protein level of ionized calcium-binding adapter molecule-1 (Iba-1), a microglial marker, was significantly increased in a time-dependent manner after CCI peaking on day 7 (Fig. 3g, i).

**Fig. 4:**
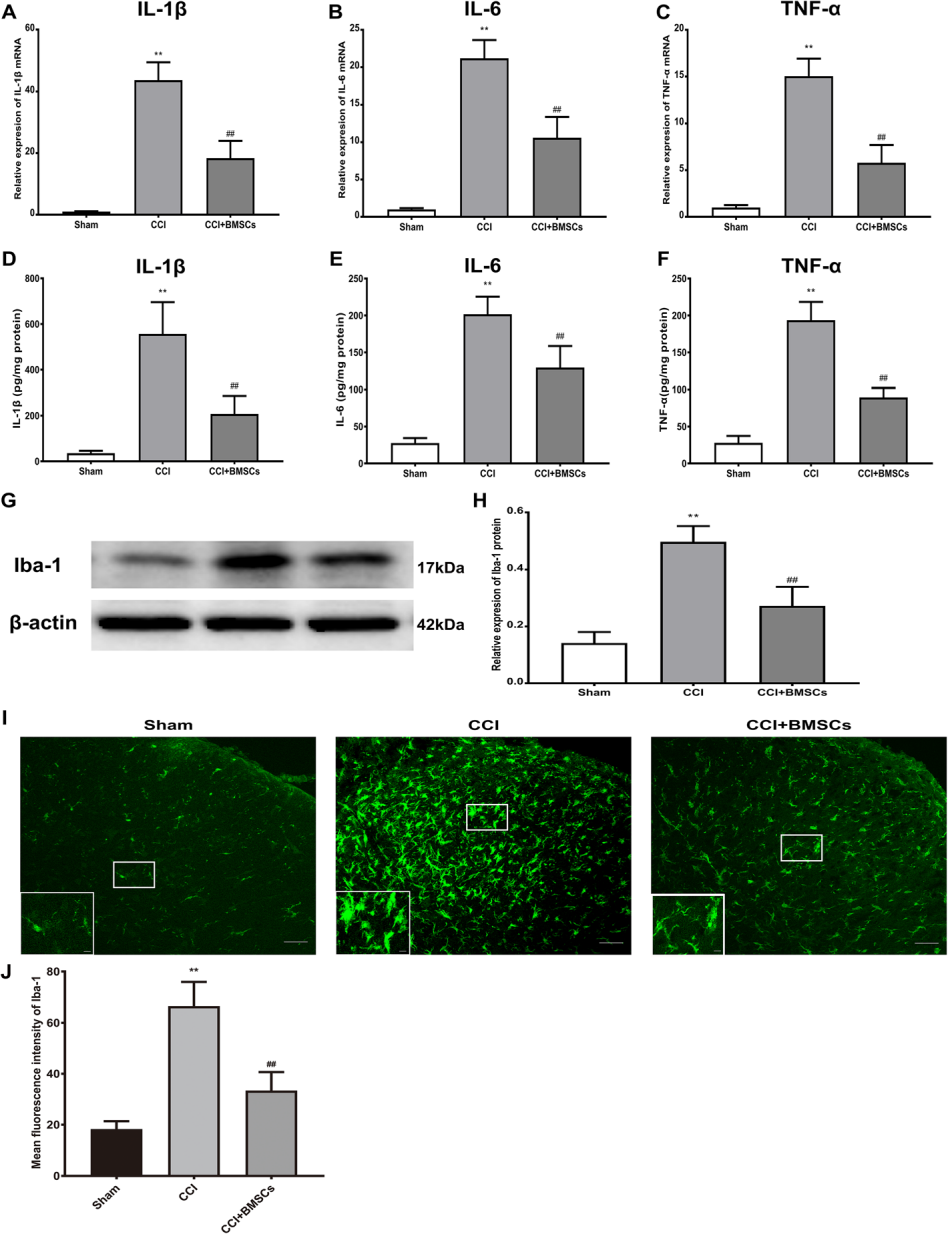
BMSCs suppressed neuropathic pain-induced production of pro-inflammatory cytokines and activation of microglia in rat spinal cord dorsal horn. **a**–**f** Real-time RT-PCR and ELISA data showed that CCI induced an increase of IL-1β, IL-6, and TNF-α in the ipsilateral spinal cord dorsal horn of rat, while intrathecal injection of BMSCs inhibited the changes of IL-1β, IL-6, and TNF-α in the rat ipsilateral spinal cord dorsal horn. **g** Representative image of protein level of Iba-1 in the rat ipsilateral spinal cord dorsal horn. **h** Quantitative analysis of western blotting results showed that CCI surgery significantly increased the expression of Iba-1, in contrast, intrathecal delivery of BMSCs decreased the expression of Iba-1 in the rat ipsilateral spinal cord dorsal horn. **i** Representative immunohistological staining showed that BMSCs inhibited the CCI-induced upregulation of microglia marker Iba-1 in the rat ipsilateral spinal cord dorsal horn. Scale bar: 50 μm; 10 μm (inserts). **j** Quantitative analysis of mean fluorescence intensity of Iba-1. The data are expressed as the means ± SD (*n* = 8 in each group). **P < 0.01 versus the sham group. ^##^*P* < 0.01 versus the CCI group. Statistical significance was determined by one-way analysis of variance (ANOVA) with post hoc Tukey test

Considering that intrathecal administration of BMSCs significantly reversed pain-like behaviors in neuropathic pain rats, we next evaluated a possible modulatory action of BMSCs on pro-inflammatory cytokines production and microglial activation. As shown in Fig. 4a–f, the mRNA and protein expression of IL-1β, IL-6, and TNF-α in the ipsilateral spinal cord dorsal horn was increased in the CCI group. In contrast, the expression of IL-1β, IL-6, and TNF-α was reduced significantly after BMSCs (5 × 10^6^) injection in the ipsilateral spinal cord dorsal horn 7 days after CCI surgery. We also found that intrathecal BMSCs (5 × 10^6^) treatment inhibited the expression of Iba-1 in the ipsilateral spinal cord dorsal horn (Fig. 4g, h). Immunohistochemical analysis also showed that Iba-1 expression was increased and that microglial cells were activated (retracted processes, increased cell body size) in the ipsilateral spinal cord dorsal horn 7 days after CCI surgery. In contrast, BMSCs (5 × 10^6^) injection significantly suppressed Iba-1 expression and inhibited the activation of microglial cells (Fig. 4i, j).

### BMSCs secreted TSG-6 to relieve neuropathic pain

**Fig. 5:**
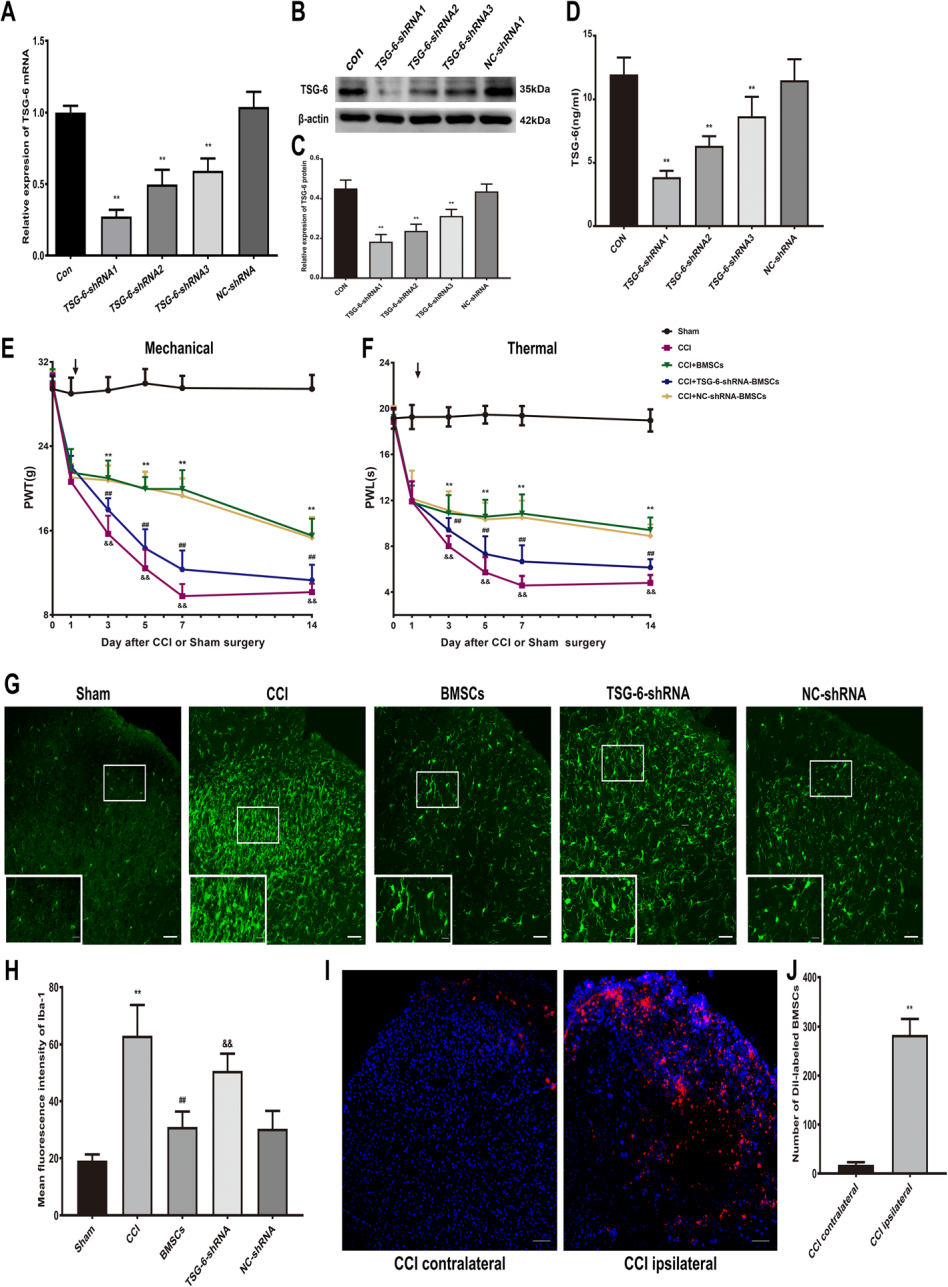
BMSCs release TSG-6 to inhibit neuropathic pain and suppress microglia activation in CCI rats. **a**–**c** The gene and protein expression of TSG-6 in control BMSCs, and in BMSCs transfected with TSG-6-shRNA1, TSG-6-shRNA2, TSG-6-shRNA3, and negative control (NC)-shRNA. **d** The ELISA analysis showed TSG-6 release in control BMSCs, and in BMSCs transfected with TSG-6-shRNA1, TSG-6-shRNA2, TSG-6-shRNA3, and negative control (NC)-shRNA. Reversal of BMSCs-induced inhibition of mechanical allodynia (**e**) and thermal hyperalgesia (**f**) in CCI rats by transfecting TSG-6-shRNA1. Arrows in (**e**, **f**) indicated the time of BMSCs injection. **g** Immunohistological staining showed that the inhibitory effect of BMSCs on microglia activation in the ipsilateral spinal cord dorsal horn was reduced after TSG-6 was knocked down. Scale bar: 50 μm; 10 μm (inserts). **h** Quantitative analysis of mean fluorescence intensity of Iba-1. **i** Localization of intrathecally injected CM-Dil-labeled BMSCs on day 3 after injection in the spinal cord dorsal horn. Scale bar: 50 μm. **j** Number of CM-Dil-labeled BMSCs in spinal cord dorsal horn. The data are expressed as the means ± SD (*n* = 8 in each group). ^**^P < 0.01 versus the sham group. ^##^*P* < 0.01 versus the CCI + BMSCs group. ^&&^*P* < 0.01 versus the BMSCs group. Statistical significance was determined by one-way analysis of variance (ANOVA) with post hoc Tukey test (**a**, **c**, **d**, **h**), two-way analysis of variance (ANOVA) with post hoc Tukey test (**e** and **f**), Student’s *t* test (two-tailed) (**j**)

The above data showed that BMSCs possess a rapid analgesic effect on CCI-induced pain behavior and a potent inhibitory effect on CCI-induced neuroinflammation. We reasoned that BMSCs exert this analgesic effect by secreting some anti-inflammatory factors. Previous studies have proven that BMSCs can exert a beneficial effect in corneal neovascularization and renal fibrosis by secreting the anti-inflammatory protein TSG-6 [44, 45]. Thus, we investigated whether BMSCs exert their therapeutic effect on CCI-induced neuropathic pain by releasing TSG-6. We knocked down the expression of TSG-6 in BMSCs by transfecting shRNA targeting TSG-6 (Fig. 5a–d). Intrathecal injection of BMSCs (5 × 10^6^) transfected with TSG-6-shRNA showed a weak analgesic effect compared with that of intrathecal injection of non-transfected BMSCs- or NC-shRNA transfected BMSCs (Fig. 5e, f). Next, we found that the inhibitory effect of BMSCs (5 × 10^6^) on microglial activation in the ipsilateral spinal cord dorsal horn was reduced after TSG-6 was knocked down, as Iba-1 immunostaining was enhanced in the ipsilateral spinal cord dorsal horn compared that of to the non-transfected BMSCs- or NC-shRNA transfected BMSCs-treated CCI rats (Fig. 5g, h).

We also observed the localization of intrathecally injected BMSCs, and we tracked Dil dye-labeled BMSCs in the spinal cord dorsal horn of CCI rats on day 3 after intrathecal injection. As shown in Fig. 5i, j, the Dil-labeled BMSCs were mainly distributed in the ipsilateral spinal cord dorsal horn on day 3 after intrathecal injection, which demonstrated that the BMSCs migrated to and survived in the ipsilateral spinal cord dorsal horn after CCI.

### Exogenous TSG-6 attenuated CCI-induced neuropathic pain and microglia activation

**Fig. 6:**
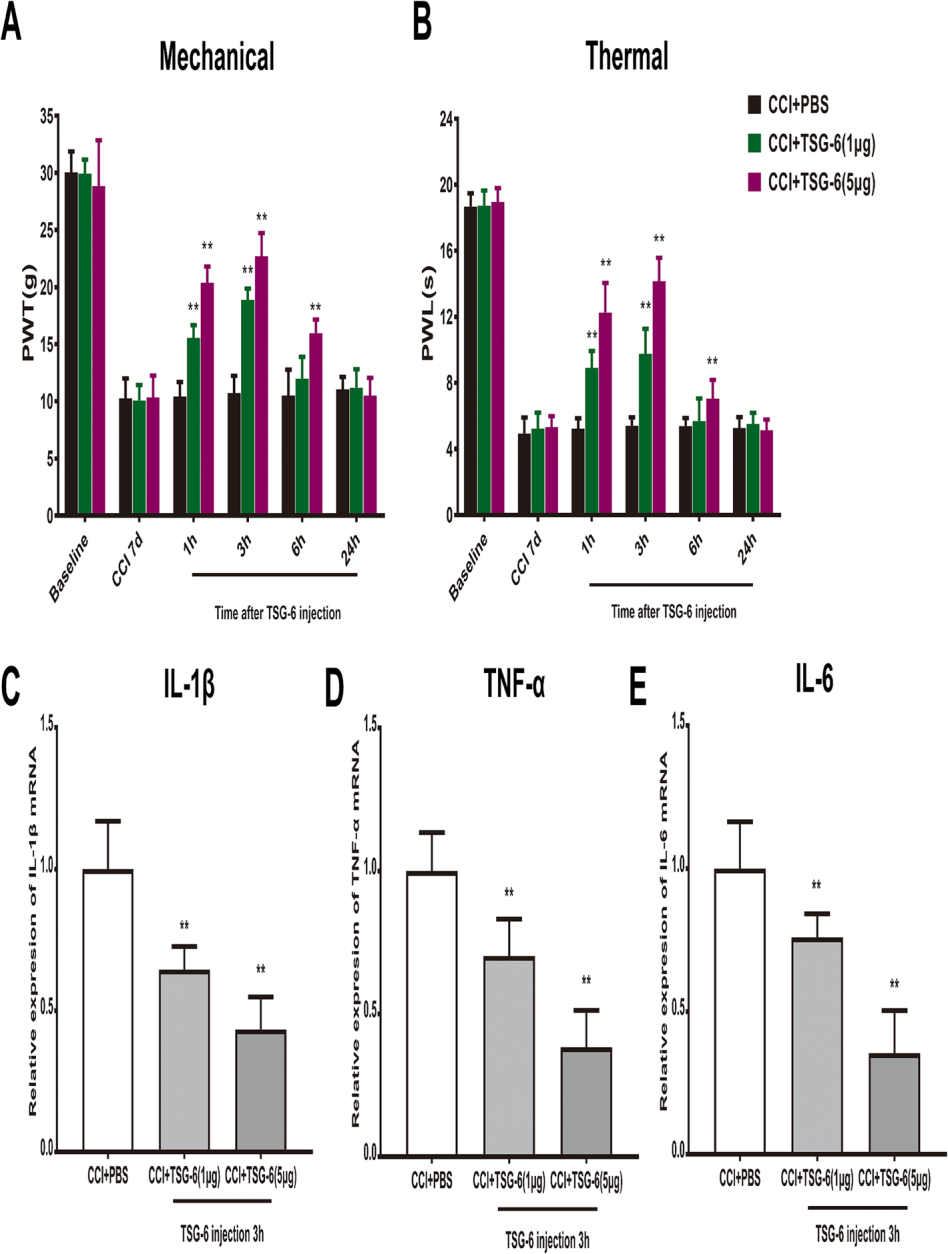
Intrathecal administration of exogenous TSG-6 attenuates CCI-induced neuropathic pain and microglia activation. Dose-dependent reversal of mechanical allodynia (**a**) and thermal hyperalgesia (**b**) by intrathecal injection of TSG-6 at 7 days after CCI. **c**–**e** Dose-dependent inhibition of CCI-induced upregulation of IL-1β, IL-6, and TNF-α after intrathecal TSG-6 injection 3 h in the ipsilateral spinal cord dorsal horn at 7 days after the CCI surgery. The data are expressed as the means ± SD (*n* = 8 in each group). **P < 0.01 versus the CCI + PBS group. Statistical significance was determined by two-way analysis of variance (ANOVA) with post hoc Tukey test (**a**, **b**), one-way analysis of variance (ANOVA) with post hoc Tukey test (**c**–**e**)

To further conform that TSG-6 is sufficient to alleviate neuropathic pain, we observed the antinociceptive effect of exogenous recombinant TSG-6 on CCI-induced mechanical allodynia and heat hyperalgesia. Two doses of recombinant TSG-6 (1 μg and 5 μg) were intrathecally delivered on day 7 after CCI and significantly decreased the withdrawal threshold and withdrawal latency in a dose-dependent manner. This therapeutic effect peaked at 3 h after TSG-6 administration (Fig. 6a, b). Next, we evaluated the inhibitory effect of exogenous TSG-6 on CCI-induced neuroinflammation. As shown in Fig. 6c–e, CCI-induced upregulation of IL-1β, IL-, and TNF-α was reduced significantly at 3 h after intrathecal injection of recombinant TSG-6 in the ipsilateral spinal cord dorsal horn at 7 days after CCI surgery.

### TSG-6 secreted by BMSCs suppressed CCI-induced neuroinflammation by inhibiting the TLR2/MyD88/NF-κB signaling pathway in spinal cord microglia

**Fig. 7:**
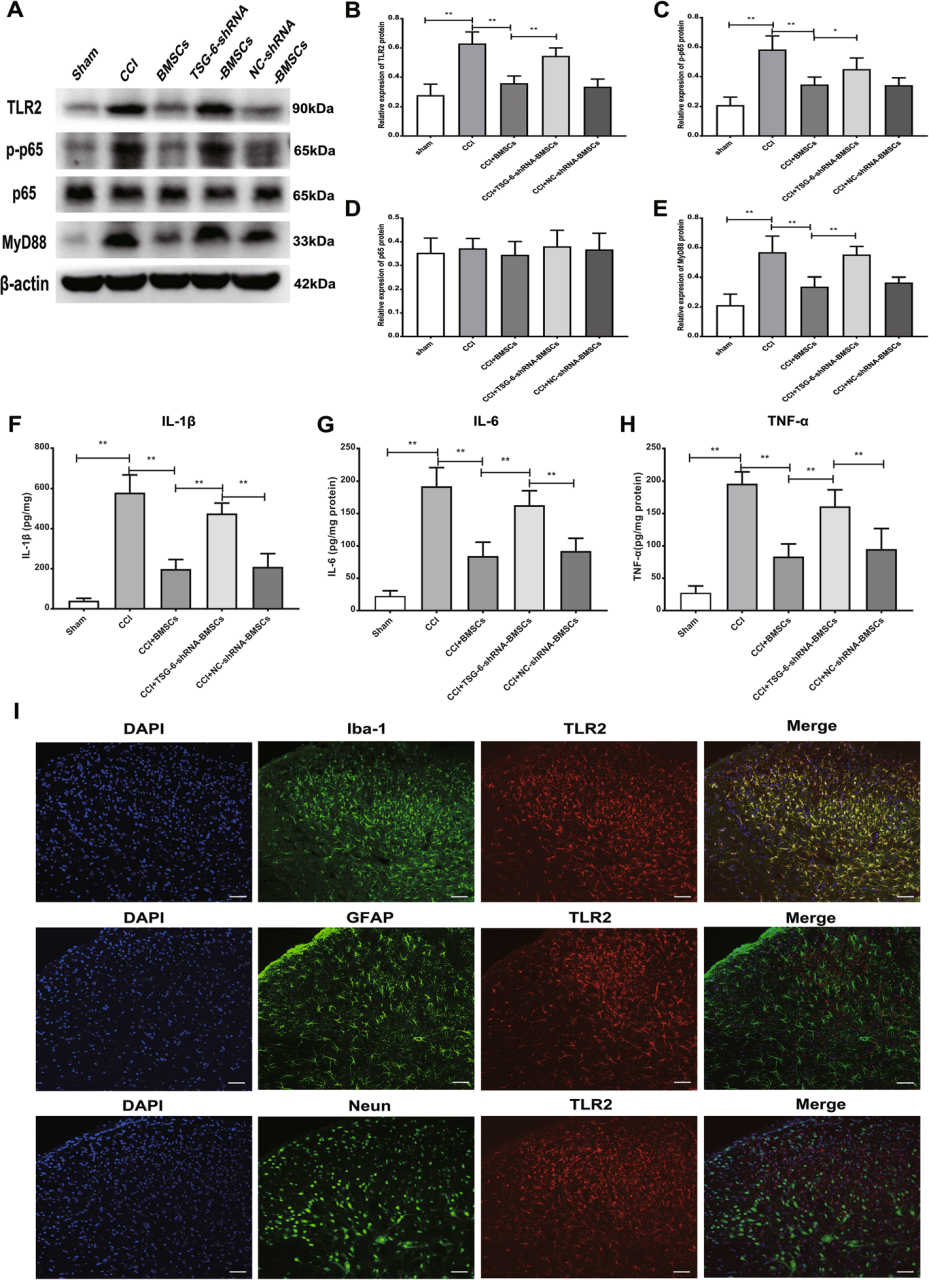
BMSCs down-regulated the TLR2/MyD88/NF-κB signaling pathway and reduced the protein levels of pro-inflammatory cytokines in CCI rats by releasing TSG-6. **a** Representative image of protein levels of TLR2, MyD88, p-p65, p65 in the ipsilateral spinal cord dorsal horn. **b**–**e** Quantitative analysis of western blotting results showed that BMSCs intrathecal delivery significantly inhibited the protein levels of TLR2, MyD88, p-p65 in the CCI rat ipsilateral spinal cord dorsal horn, in contrast, the TSG-6-shRNA transfection abrogated the inhibitory effect of BMSCs on the expression of these proteins in the CCI rat ipsilateral spinal cord dorsal horn. BMSCs intrathecal injection significantly reduced the protein contents of IL-1β (**f**), IL-6 (**g**), and TNF-α (**h**) in the CCI rat ipsilateral spinal cord dorsal horn, while the inhibitory effect of BMSCs on the production of these pro-inflammatory cytokines was weakened after TSG-6 was knock down. **i** TLR2 immunosignals were co-localized with signals of Iba-1-positive microglia, but not with signals of GFAP-positive astrocytes or NeuN-positive neurons in the CCI rat ipsilateral spinal cord dorsal horn. Scale bar: 50 μm. The data are expressed as the means ± SD (*n* = 8 in each group). *P < 0.05, **P < 0.01. Statistical significance was determined by one-way analysis of variance (ANOVA) with post hoc Tukey test

Previous studies have demonstrated that the anti-inflammatory protein TSG-6 secreted by BMSCs can inhibit the activation of the TLR2/MyD88/NF-κB signaling pathway [27, 40]. In addition, it has been proven that TLR2 plays a key role in nerve injury-induced spinal cord glial cell activation and is necessary for the development of neuropathic pain [23]. To determine whether TSG-6 secreted by BMSCs exerts its analgesic effect by inhibiting the TLR2/MyD88/NF-κB signaling pathway in the spinal cord dorsal horn, we explored the levels of TLR2, MyD88, phosphorylated NF-κB p65, and total NF-κB p65 by Western blotting. As expected, compared with the basal protein levels of TLR2, MyD88, and p-p65 in the sham group, the protein expression of TLR2, MyD88, and p-p65 in the ipsilateral spinal cord dorsal horn was increased in CCI rats; in contrast, the CCI-induced upregulation of TLR2, MyD88, and p-p65 was inhibited after intrathecal delivery of BMSCs (5 × 10^6^). However, TSG-6-shRNA transfection abrogated the inhibitory effect of BMSCs on the expression of these proteins in the ipsilateral spinal cord dorsal horn. In addition, NC-shRNA transfection did not affect the inhibitory effect of BMSCs (Fig. 7a–e). Next, we measured the protein levels of pro-inflammatory cytokines in the ipsilateral spinal cord dorsal horn. The results showed that the protein levels of IL-1β, IL-6, and TNF-α were dramatically increased in the CCI group compared with the sham group. Intrathecal injection of BMSCs (5 × 10^6^) significantly decreased the protein levels of IL-1β, IL-6, and TNF-α compared with those in the CCI group; however, the anti-inflammatory effect of BMSCs was abrogated when the expression of TSG-6 was suppressed by TSG-6-shRNA (Fig. 7f–h). Given that neuropathic pain can induce the activation of the TLR2/MyD88/NF-κB signaling pathway, we continued to examine the cellular distribution of TLR2 in the spinal cord dorsal horn after CCI by immunofluorescence staining. We performed double staining for TLR2 and three major spinal cord cell specific markers: NeuN (a neuronal marker), GFAP (an astrocyte marker), and Iba-1(a microglial marker). As shown in Fig. 7i, TLR2-immunoreactive (IR) mainly co-localized with Iba-1-IR microglia but not with GFAP-positive astrocytes, or NeuN-positive neurons. These results suggested that TLR2 was expressed exclusively in microglia after CCI surgery in the spinal cord dorsal horn.

### TLR2 stimulation induced the activation of primary microglia and the production of pro-inflammatory cytokines

**Fig. 8:**
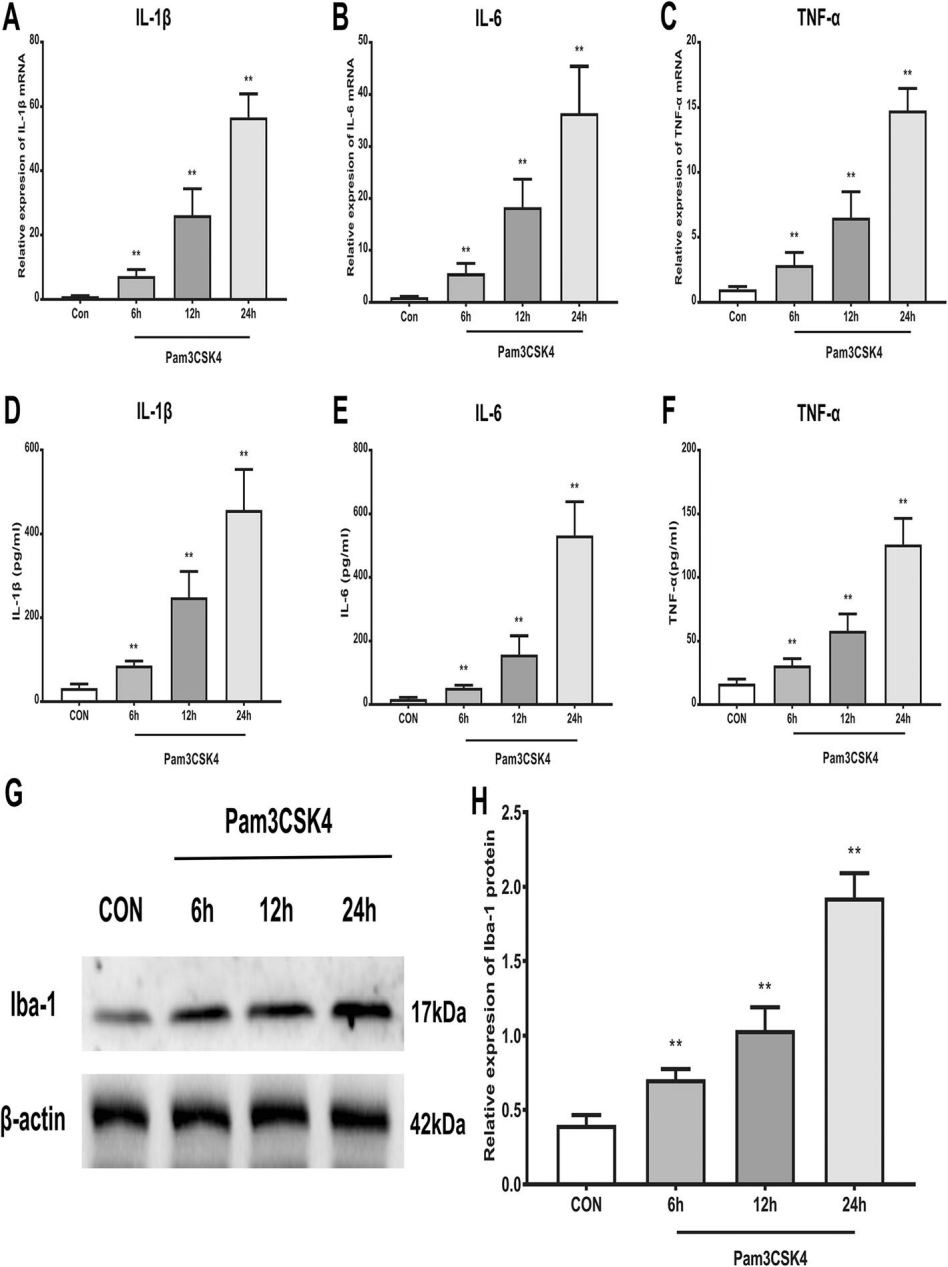
TLR2 triggered the activation of primary microglia and enhanced the production of pro-inflammatory cytokines. **a**–**f** TLR2 specific agonist Pam3CSK4 (500 ng/ml) induced a time-dependent up-regulation of mRNA and protein expression of IL-1β, IL-6, and TNF-α in primary microglia. **g** Representative image of protein level of Iba-1 in Pam3CSK4-treated primary microglia. **h** Quantitative analysis of western blotting result showed that Pam3CSK4 significantly increased the protein expression of Iba-1 in primary microglia in a time-dependent manner. The data are expressed as the means ± SD (*n* = 8 in each group). **P < 0.01 versus the control group. Statistical significance was determined by one-way analysis of variance (ANOVA) with post hoc Tukey test

Primary microglia were cultured from the rat cerebral cortex and then treated with the specific TLR2 agonist Pam3CSK4 to further investigate TLR2-mediated microglial activation. As shown in Fig. 8a–f, the exposure of primary microglia to Pam3CSK4 (500 ng/ml) induced a time-dependent upregulation of the mRNA and protein expression of pro-inflammatory cytokines, including IL-1β, IL-6, and TNF-α, compared with the level in the control group. Furthermore, Western blot analysis also showed that Pam3CSK4 treatment increased the protein level of Iba-1 in primary microglia (Fig. 8g, h). These data demonstrated that TLR2 stimulation induced the activation of primary microglia and the production of pro-inflammatory cytokines.

### TSG-6 released from BMSCs suppressed pro-inflammatory cytokine production through the TLR2/MyD88/NF-κB signaling pathway in primary microglia

**Fig. 9:**
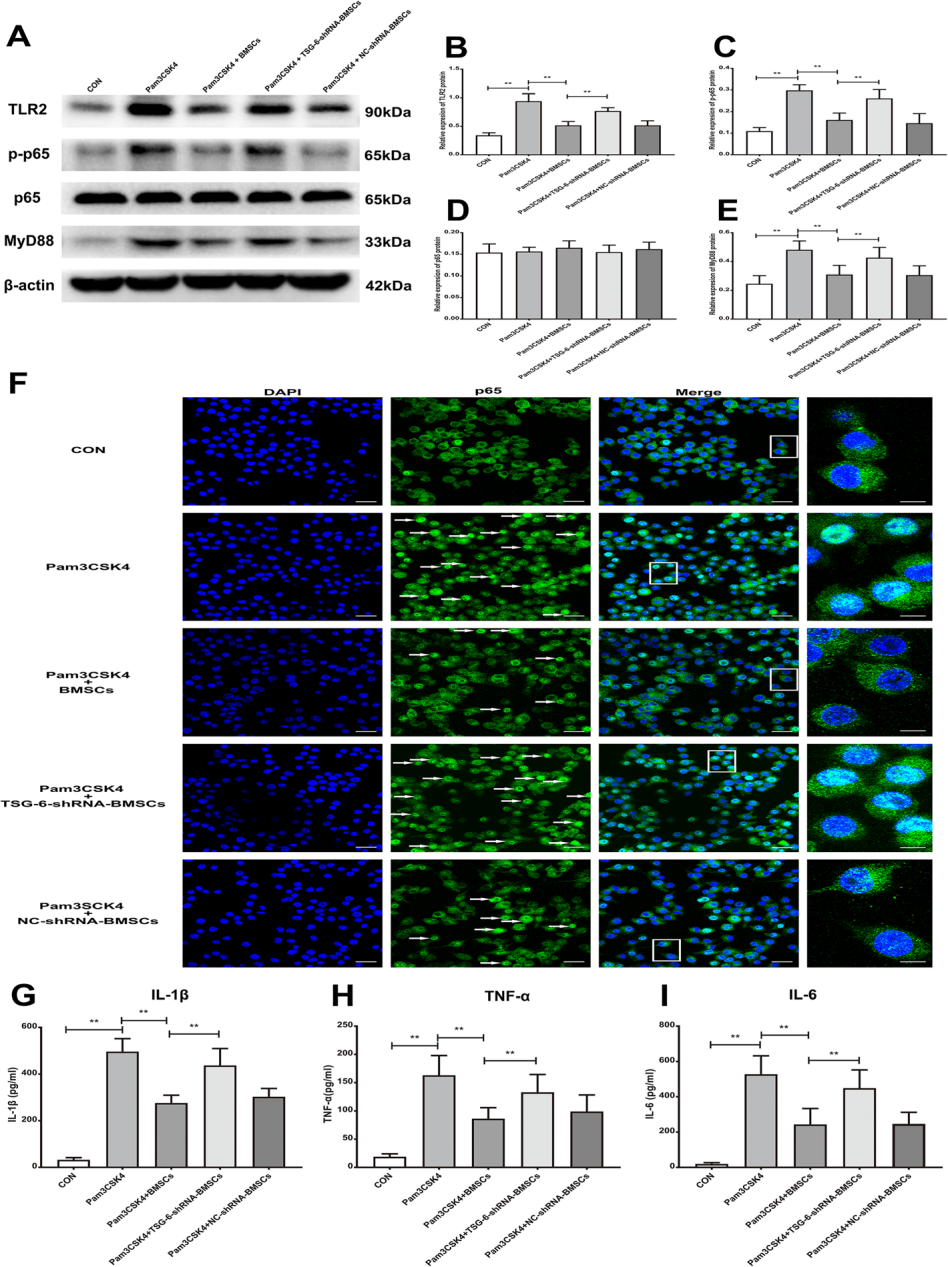
BMSCs released TSG-6 to reduce pro-inflammatory cytokine production through TLR2/MyD88/NF-κB signaling pathway in primary microglia. **a** Representative image of protein levels of TLR2, MyD88, p-p65, p65 in primary microglia. **b**–**e** Quantitative analysis of western blotting result showed that BMSCs co-culture inhibited the Pam3CSK4-induced the protein expression of TLR2, MyD88, p-p65 in primary microglia, in contrast, the inhibitory effect of BMSCs on the expression of these proteins was weakened after TSG-6 was knock down. **f** Typical micrographs of immunocytochemical staining are shown for the cytoplasmic and nuclear distribution of NF-κB p65. Scale bar: 30 μm; 10 μm (magnified graphs). **g**–**i** ELISA analysis showing BMSCs co-culture inhibited IL-1β, IL-6, and TNF-α release in Pam3CSK4-treated primary microglia; however, the anti-inflammatory effect of BMSCs was compromised after TSG-6-shRNA treatment. The data are expressed as the means ± SD (*n* = 8 in each group). **P < 0.01. Statistical significance was determined by one-way analysis of variance (ANOVA) with post hoc Tukey test

To further confirm that TSG-6 secreted by BMSCs inhibits microglial activation and suppresses pro-inflammatory cytokine production through the TLR2/MyD88/NF-κB signaling pathway, we performed co-culture experiments. Pam3CSK4-treated primary microglia were co-cultured with BMSCs for 24 h. Compared with the basal protein levels of TLR2, MyD88, and p-p65 in the control group, the levels of these proteins were upregulated in Pam3CSK4-treated primary microglia. In contrast, the Pam3CSK4-induced upregulation of TLR2, MyD88, and p-p65 in primary microglia was inhibited after co-culture with BMSCs (5 × 10^6^). However, BMSCs transfected with TSG-6-shRNA had no significant inhibitory effect on the TLR2/MyD88/NF-κB signaling pathway compared with non-transfected BMSCs or NC-shRNA transfected BMSCs (Fig. 9a–e). Next, we analyzed NF-κB p65 nuclear translocation in primary microglia using immunofluorescence staining. In the control group, NF-κB p65 largely remained in the cytoplasm. After Pam3CSK4 treatment, NF-κB p65 translocated to the nucleus in primary microglia, but to a lesser extent in the BMSCs (5 × 10^6^) co-culture group, in contrast, TSG-6 knock down significantly attenuated the inhibitory effect of BMSCs on NF-κB p65 nuclear translocation in Pam3CSK4-stimulated microglia (Fig. 9f). We also examined the effect of BMSCs on the release of pro-inflammatory cytokines by primary microglia. As indicated in Fig. 9g–i, the secretion of IL-1β, IL-6, and TNF-α in Pam3CSK4-treated microglia were significantly increased compared with control microglia. As expected, BMSCs (5 × 10^6^) significantly inhibited IL-1β, IL-6, and TNF-α secretion in microglia induced by Pam3CSK4; however, the anti-inflammatory effect of BMSCs was compromised after TSG-6-shRNA treatment.

### Exogenous TSG-6 reduced pro-inflammatory cytokine production by inhibiting the TLR2/MyD88/NF-κB signaling pathway in primary microglia

**Fig. 10:**
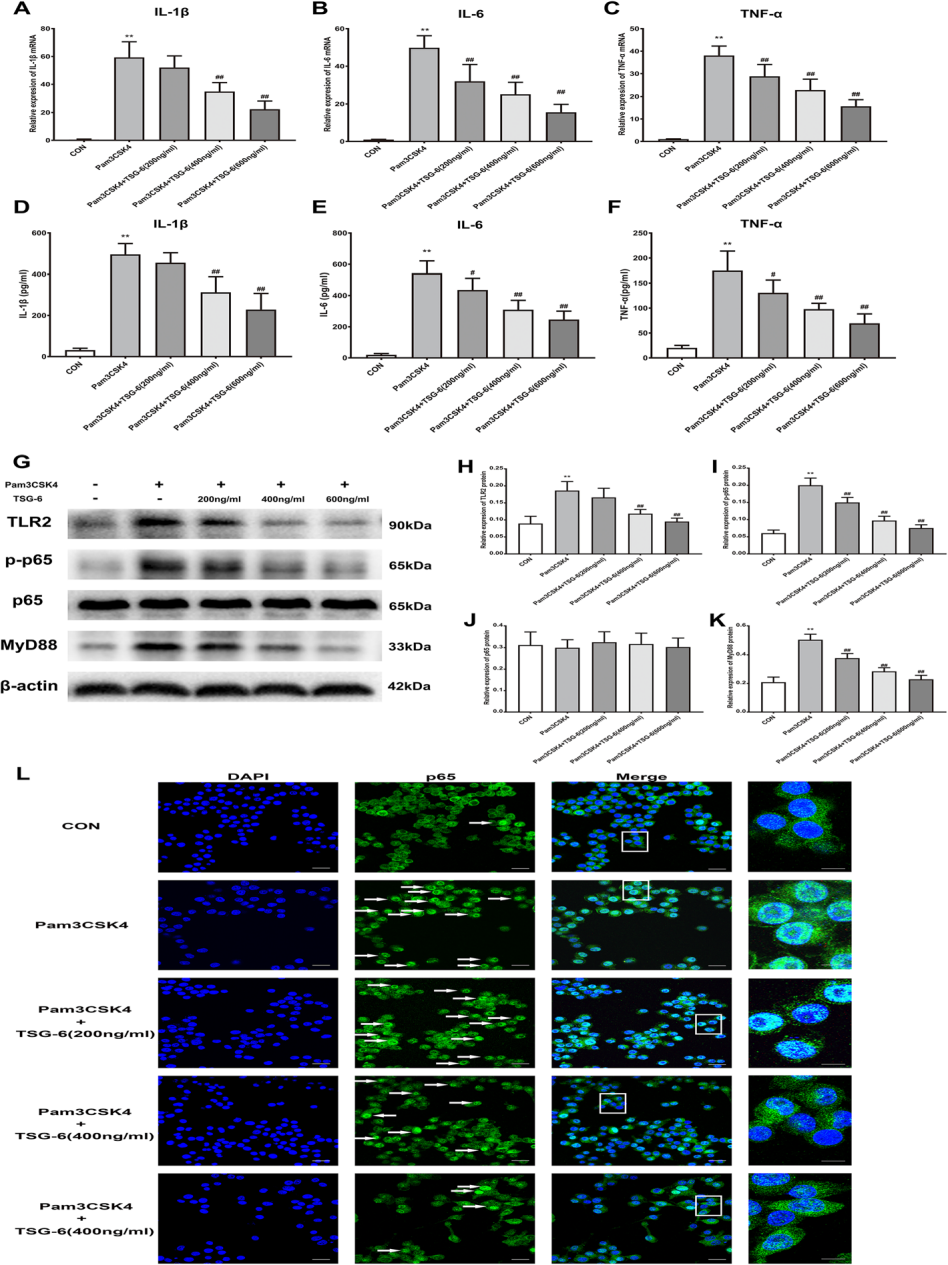
Exogenous TSG-6 inhibited the expression of pro-inflammatory cytokines and suppressed the TLR2/MyD88/NF-κB signaling pathway in Pam3CSK4-treated primary microglia. **a**–**c** Recombinant TSG-6 inhibited the increased mRNA levels of IL-1β, IL-6, and TNF-α in Pam3CSK4-treated primary microglia in a concentration-dependent manner. **d**–**f** Recombinant TSG-6 inhibited the release of IL-1β, IL-6, and TNF-α in Pam3CSK4-treated primary microglia in a concentration-dependent manner. **g** Representative image of protein levels of TLR2, MyD88, p-p65, p65 in primary microglia. **h**–**k** Quantitative analysis of the Western blot result showed that recombinant TSG-6 inhibited the Pam3CSK4-induced up-regulation of TLR2, MyD88, p-p65 protein expression in primary microglia in a concentration-dependent manner. **l** Typical micrographs of immunocytochemical staining are shown for the cytoplasmic and nuclear distribution of NF-κB p65. Scale bar: 30 μm; 10 μm (magnified graphs). The data are expressed as the means ± SD (*n* = 8 in each group). **P < 0.01 versus the control group. ^#^*P* < 0.05, ^##^*P* < 0.01 versus the Pam3CSK4 group. Statistical significance was determined by one-way analysis of variance (ANOVA) with post hoc Tukey test

We examined the effect of exogenous recombinant TSG-6 on the expression of pro-inflammatory cytokines in Pam3CSK4-stimulated primary microglia. As shown in Fig. 10a–f, both the mRNA expression and the protein contents of IL-1β, IL-6, and TNF-α were upregulated in Pam3CSK4-stimulated microglia compared with control microglia; however, recombinant TSG-6 significantly decreased the mRNA and the protein levels of IL-1β, IL-6, and TNF-α in a concentration-dependent manner. We also explored the effect of exogenous TSG-6 on the TLR2/MyD88/NF-κB signaling pathway in primary microglia. We added recombinant TSG-6 (200 ng/ml, 400 ng/ml, or 600 ng/ml) to the primary microglial culture medium during Pam3CSK4 treatment. The results showed that the protein expression of TLR2, MyD88, and p-p65 in primary microglia was inhibited in a concentration-dependent manner when Pam3CSK4-treated microglia were cultured with recombinant TSG-6 (Fig. 10g–k). Finally, we analyzed NF-κB p65 nuclear translocation in primary microglia. In the control group, NF-κB p65 largely remained in the cytoplasm. After Pam3CSK4 treatment, NF-κB p65 translocated to the nucleus in primary microglia, in contrast, TSG-6 treatment significantly inhibited the NF-κB p65 nuclear translocation in Pam3CSK4-stimulated microglia in a concentration-dependent manner (Fig. 10l).

## Discussion

Increasing evidence has demonstrated that transplantation of BMSCs consistently alleviates neuropathic pain [1, 34, 35]. However, the underlying mechanism of the analgesic effect of BMSCs transplantation remains to be determined. In the present study, we found that intrathecal transplantation of BMSCs alleviated CCI-induced allodynia and hyperalgesia and reduced the expression of pro-inflammatory cytokines, such as IL-1β, IL-6, and TNF-α, in the ipsilateral spinal cord dorsal horn after CCI surgery, possibly through the secretion of the anti-inflammatory protein TSG-6. In addition, our results also showed that intrathecal delivery of exogenous recombinant TSG-6 potently inhibited neuropathic pain after CCI. Furthermore, we demonstrated that TSG-6 secreted by BMSCs exerted an antinociceptive effect, at least partly by suppressing the activation of the TLR2/MyD88/NF-κB signaling pathway in spinal cord dorsal horn microglia in vivo and in vitro. To our knowledge, the current research is the first to demonstrate that TSG-6 secreted by BMSCs exerts an analgesic effect via inhibition of the TLR2/MyD88/NF-κB signaling pathway in spinal cord dorsal horn microglia.

In this study, our data showed that intrathecal injection of BMSCs significantly ameliorated mechanical allodynia and hyperalgesia in CCI rats. The antinociceptive effect of BMSCs peaked on day 7 post-CCI and lasted for at least 14 days. Consistent with our results, previous studies demonstrated that intrathecal delivery of BMSCs can relieve neuropathic pain and that the analgesic effect of BMSCs persists for at least 14 days [34, 35]. Mesenchymal stem-cell therapy, such as BMSCs treatment, has been considered a potentially successful approach for treating various diseases including neuropathic pain [37, 46], spinal cord injury [47, 48], Alzheimer’s disease [49, 50], stroke [51, 52], and lung injury [53, 54]. BMSCs were the earliest and the most widely studied MSCs in vitro and in vivo. Initially, studies tested the homing characteristic of BMSCs to the injury site and the potential of BMSCs to repair damaged tissues by differentiating into the appropriate cell type. Recently, an increasing number of studies have demonstrated that the protective effects of BMSCs might be due to their potent immunomodulatory and immunosuppressive properties via paracrine secretions [55, 56]. In the present study, we also found that BMSCs inhibited the expression of pro-inflammatory cytokines and the activation of microglia in the spinal cord dorsal horn by secreting soluble factors. TSG-6 is a pleiotropic immunomodulatory molecule activated rapidly in response to stimulation by several pro-inflammatory cytokines and functions at an early phase of the inflammatory process [38, 57, 58]. Several lines of evidence suggest that TSG-6 secreted by MSCs can produce anti-inflammatory effects in a wide variety of diseases, such as bronchopulmonary dysplasia [59], diabetic retinopathy [60], corneal injury [44], inflammatory bowel disease [39], renal fibrosis [45], severe acute pancreatitis [61], and other inflammation-associated diseases. In the present study, we showed that intrathecal injection of BMSCs reduced the expression of IL-1β, IL-6, and TNF-α in the ipsilateral spinal cord dorsal horn; meanwhile, BMSCs administration alleviated CCI-induced allodynia and hyperalgesia. However, when we used specific TSG-6-shRNA to knock down the expression of the TSG-6 gene in BMSCs, the protective effects of BMSCs on pro-inflammatory cytokines and pain behaviors compared with those of non-transfected BMSCs or NC-shRNA transfected BMSCs were partly weakened. To further confirm the key role of TSG-6 in ameliorating neuroinflammation, we intrathecally injected exogenous recombinant TSG-6 protein directly to the CCI rats. The recombinant TSG-6 protein also reduced the expression of IL-1β, IL-6, and TNF-α in the ipsilateral spinal cord dorsal horn and exerted an analgesic effect on the CCI-induced nociceptive response in a dose-dependent manner. These results demonstrated that TSG-6 made a major contribution to the BMSCs-mediated inhibition of neuroinflammation and neuropathic pain.

Microglia are resident immune cells and act as the main form of active immune defense in the CNS. Accumulating evidence has indicated that peripheral nerve injury induced microgliosis in the ipsilateral spinal cord dorsal horn is a major contributor to neuropathic pain [62, 63]. In this study, we found that activated microglia in CCI rats displayed a hypertrophied or amoeboid morphology, which was indicative of microglial activation, compared with the resting, ramified morphology of microglia in the sham group. However, intrathecally injected BMSCs caused a decrease in the number of microglia and reduced the expression of the microglial marker Iba-1. These findings suggested that BMSCs inhibited the activation of microglia in the ipsilateral spinal cord dorsal horn following peripheral nerve injury.

TLRs are type I transmembrane receptors that play a key recognition role in the innate immune response. Previous studies have found that TLR2 is involved in microglial activation in a variety of neurological diseases and psychiatric disorders, such as Parkinson’s disease [64], spinal cord injury [65], Alzheimer’s disease [66], multiple sclerosis [67], and repeated social defeat stress [68]. In addition, TLR2 also contributes to nerve injury-induced spinal cord microglial activation and subsequent neuropathic pain [23]. Consistent with previous observations, we found that the protein level of TLR2 was increased in the ipsilateral spinal cord dorsal horn after CCI surgery and that TLR2 was mainly expressed in ipsilateral spinal cord dorsal horn microglia. It has been demonstrated that TSG-6 secreted by BMSCs can inhibit the activation of the TLR2/MyD88/NF-κB signaling pathway [27, 40]. Thus, it can be reasoned that TSG-6 secreted by BMSCs can inhibit the activation of the TLR2/MyD88/NF-κB signaling pathway in ipsilateral spinal cord dorsal horn microglia. Our results suggested that the upregulation of TLR2, MyD88, and p-p65 in the ipsilateral spinal cord dorsal horn after CCI surgery was inhibited after intrathecal injection of BMSCs or intrathecal administration of recombinant TSG-6; however, the inhibitory effect of BMSCs on these proteins was weakened when the expression of TSG-6 was knocked down. In addition, our in vitro experiments further proved that TSG-6 inhibited the activation of the TLR2/MyD88/NF-κB signaling pathway in primary microglia.

TLR2 induces MyD88-dependent signaling pathways to activate NF-κB, which increases the production of pro-inflammatory molecules, such as pro-inflammatory cytokines (TNF-α, IL-1β, and IL-6). Under unstimulated conditions, NF-κB is localized in the cytoplasm and is bound to the inhibitory protein IκB. After stimulation, specific kinases phosphorylate IκB, causing its rapid degradation by the proteasome and subsequently allowing NF-κB to translocate into the nucleus, where it binds to specific sequences in the promoter regions of target genes [69]. To further elucidate the molecular mechanism underlying the anti-inflammatory effect of TSG-6 in activated microglia, we investigated the nuclear translocation of p65. Our results showed that BMSCs co-culture substantially suppressed Pam3CSK4-induced nuclear translocation of p65 in primary microglia and this inhibitory effect of BMSCs on p65 nuclear translocation in Pam3CSK4-treated microglia was significantly attenuated after TSG-6 knock down.

Increasing evidence has shown that neuroinflammation plays an important role in the development and progression of nerve injury-induced neuropathic pain [70, 71]. As a critical cellular component of innate immunity in the CNS, activated microglia can secrete a variety of pro-inflammatory cytokines, such as IL-1β, IL-6, and TNF-α, that contribute to neuropathic pain by activating nociceptive neurons [14]. Thus, inhibition of the production of pro-inflammatory cytokines in microglia can attenuate neuropathic pain. In the present study, we used ELISA assays to investigate the effects of BMSCs and TSG-6 on pro-inflammatory cytokine production in the ipsilateral spinal cord dorsal horn of CCI rats and Pam3CSK4-treated primary microglia. Our data demonstrated that BMSCs markedly decreased the mRNA and protein levels of IL-1β, IL-6, and TNF-α in the ipsilateral spinal cord dorsal horn of CCI rats via the secretion of TSG-6. Our in vitro experiments also proved that TSG-6 secreted by BMSCs inhibited Pam3CSK4-induced pro-inflammatory secretion in primary microglia.

In the present study, we found that a single intrathecal injection of the recombinant TSG-6 protein can significantly inhibit CCI-induced neuropathic pain about 24 h, but intrathecal administration of BMSCs remarkably alleviated CCI-induced mechanical allodynia and thermal hyperalgesia lasted for at least 14 days. Injection of recombinant human (rh) TSG-6 into the tail veins of mice has indicated a short half-life in plasma [72]. Thus, we speculated that the short-term analgesic effect of recombinant TSG-6 protein is due to the rapid degradation. Besides, numerous studies have demonstrated that the production and secretion of TSG-6 in BMSCs is conditioned by pro-inflammatory cytokines, such as TNF-α [27, 73]. This suggested that BMSCs can sense and respond to an inflammatory microenvironment. In the present research, we have found that peripheral nerve injury-induced microglia activation in the spinal cord dorsal horn can produce and release inflammatory mediators including TNF-α. Therefore, the intrathecal administered BMSCs may continuously release TSG-6 in response to the nerve injury induced neuroinflammation in the spinal cord dorsal horn and produce sustained neuropathic pain relief.

## Conclusion

In summary, our findings demonstrated that intrathecally injected BMSCs produced sustained analgesic effects on neuropathic pain in rats via TSG-6 secretion. The present research confirmed that TSG-6 released from BMSCs significantly inhibited the nerve injury-induced activation of the TLR2/MyD88/NF-κB signaling pathway and reduced the production of the pro-inflammatory cytokines IL-1β, IL-6, and TNF-α in spinal cord dorsal horn microglia by secreting the anti-inflammatory protein TSG-6. In addition, our study proved that intrathecal injection of exogenous recombinant TSG-6 may be an effective treatment option for CCI-induced neuropathic pain.

## Data Availability

The datasets used and analyzed during the current study are available from the corresponding author on reasonable request.

## Abbreviations

BMSCs: Bone marrow mesenchymal stem cells
CCI: Chronic constriction injury
CNS: Central nervous system
IL-1β: Interleukin-1β
MyD88: Myeloid differentiation factor-88 adaptor protein
TLRs: Toll-like receptors
TNF-α: Tumor necrosis factor-α
TSG-6: TNF-α-stimulated gene 6 protein
